# Extracorporeal cardiopulmonary resuscitation versus conventional CPR in cardiac arrest: be aware of the temporal selection bias

**DOI:** 10.1186/s13054-024-04907-1

**Published:** 2024-04-18

**Authors:** Romain Jouffroy, Anne-Cécile Vie, Benoît Vivien

**Affiliations:** 1 Service de Médecine Intensive Réanimation, Hôpital Universitaire Ambroise Paré, Assistance Publique - Hôpitaux de Paris, and Paris Saclay University, Boulogne, France; 2 SAMU de Paris, Service d’Anesthésie Réanimation, Hôpital Universitaire Necker - Enfants Malades, Assistance Publique - Hôpitaux de Paris, and Paris Cité University, Paris, France

To the editor

We read with great interest the recent meta‑analysis and trial sequential analysis published in the Journal by Low et al. [1] reporting the benefit of extracorporeal cardiopulmonary resuscitation (ECPR) for both in-hospital cardiac arrest (IHCA) and out-of-hospital cardiac arrest (OHCA) outcomes improvement.

However, we believe that few issues deserve the results’ interpretation and manuscript’ conclusions.

First, the intrinsic OHCA and IHCA prognoses are clearly different [2]. While OHCA has been more studied than IHCA, their chain of survival are different and, OHCA outcome is better than IHCA [2]. Consequently, it is surprising to mix IHCA and OHCA in a meta-analysis. All the more since in Low et al. study [1], the IHCA prognosis was assessed in 4 studies out of 13, representing approximately 150 patients out of the 14,048 patients analyzed, i..e. nearly 1% of all patients.

In addition, the studies included in this meta‑analysis were mainly, but not exclusively, carried out after 2015, including the study by Kim et al. [3] representing approximately 50% of the patients. Before 2015, ECPR eligibility criteria were not the priority in the studies, which were mainly feasibility studies. Since 2015, the experts recommended the definition of criteria, i.e. eligibility criteria, in order to select patients with the best chance of success and to avoid futile ECPR implementation [2]. Among the eligibility criteria, a shockable rhythm is in itself associated with a better prognosis [4]. Thus, after 2015, ECPR studies concern selected patients, i.e. those with the highest expected success rate, that consequently directly impacts both studies’ design and studies’ results [5].

Beyond these limitations, we agree that ECPR should be considered in both IHCA and OHCA among “selected” patients, i.e. those whose prognosis is, a priori, favorable and who will benefit most from ECPR implementation.

## Data Availability

Not applicable.
